# The role of arthroscopy in the dysplastic hip—a systematic review of the intra-articular findings, and the outcomes utilizing hip arthroscopic surgery

**DOI:** 10.1093/jhps/hnv071

**Published:** 2016-01-09

**Authors:** Suenghwan Jo, Sang Hong Lee, Sung Il Wang, Bjorn Smith, John O’Donnell

**Affiliations:** 1. Department of Orthopedic Surgery, Hip Arthroscopy Australia, 21 Erin Street, Melbourne, VIC, Australia,; 2. Department of Orthopedic Surgery, Chosun University Hospital, Gwangju, South Korea and; 3. Department of Orthopedic Surgery, Chunbuk University Hospital, Jeonju, South Korea

## Abstract

Acetabular dysplasia is one of the most common sources of hip arthritis. With the recent innovation in hip arthroscopy, the question has been raised whether arthroscopy can be used to treat dysplastic hip conditions. The purposes of this systematic review are (i) describe the prevalence of intra-articular pathologies and (ii) report the outcomes of dysplastic hip treatment with hip arthroscopy as a sole treatment. Medical databases were searched for articles including arthroscopic findings and treatment of dysplastic hip with predetermined criteria. PubMed, Ovid database and CINAHL (Cumulative Index to Nursing and Allied Health Literature) were searched up until 7 January 2015. Two reviewers independently assess the eligibility of retrieved studies using titles, abstracts and full-text articles. Thirteen studies were eligible to be included for the systematic review. Overall, labral tear was the most common pathology with a prevalence rate of 77.3%. All of the four studies describing arthroscopic treatment for only borderline dysplasia reported favorable outcome. With regard to more severely dysplastic hips, two out of three studies reported acceptable outcomes while one study reported negative results. This review indicates that intra-articular pathology is commonly observed in symptomatic dysplastic hips with a labral tear being the most common pathology. Arthroscopic treatment of borderline dysplasia could provide benefits whereas treatment of more dysplastic hips is controversial. Nevertheless, there is a lack of evidence for using arthroscopy alone in hips with a center edge angle <20°. Level IV, systematic review of Level IV studies.

## INTRODUCTION

Developmental dysplasia of the hip is one of the most common reasons leading to symptomatic hip arthritis [1]. Various methods have been proposed to minimize the problems potentially resulting from abnormal hip joint contact pressure generated by the lack of femoral head coverage. The morphology and the organization of the acetabulum, which is typically shallow and anteverted, and the femoral head which is lateralized, can result in abnormally high contact pressure at the articular margin of the acetabulum [2–8]. The resultant altered hip biomechanics can cause labral tears, chondral lesions, ligamentum teres (LT) injuries, hip instability or impingement [2, 9–12]. This can ultimately lead to degenerative hip disease that may necessitate joint replacement [13–16]. Depending on the symptoms and the degree of dysplasia, treatment can be varied. In the recalcitrant conditions with moderate or severe dysplasia, the recommended definitive treatment involves reconstructing the acetabular roof. Previous literature has reported promising outcomes when the patient is treated with appropriate pelvic osteotomy procedure but the surgical procedure is associated with a high morbidity and have been reported to cause number of complications [17]. With the recent advances in hip arthroscopy in terms of instrumentation and techniques, the treatment of intra-articular pathologies which once was believed to require open surgery can be now performed with minimal incision and free of muscle and tendon damage.

The idea of treating hip conditions with arthroscopy in dysplastic hip has been negatively viewed based on the concept that the contact pressure is directed toward the lateral edge of the acetabulum. This in turn will cause early degeneration if the underlying bone structure is uncorrected. However, there is a paucity of literature on how the arthroscopic treatment will affect the dysplastic hip and the current concept is mostly derived from expert opinions based on biomechanics studies.

Thus, in this study, we aim to discuss the role of hip arthroscopy in the dysplastic hip by (i) describing the prevalence and location of pathologic lesions diagnosed with arthroscopy and (ii) reporting the outcome using arthroscopic treatment as a sole treatment. Through this process, we expect to better define the indication and contra-indication for utilizing arthroscopic treatment for symptomatic dysplastic hips.

## MATERIALS AND METHODS

The online database search was made on 23 November 2014, with keywords ‘dysplastic’, ‘dysplasia’, ‘DDH’, ‘under coverage’, ‘hypoplasia’, ‘hip’, ‘acetabular’, ‘arthroscopy’ and ‘osteotomy’ using PubMed, Ovid database (including EMBASE, Medline and Cochrane library), and CINAHL (Cumulative Index to Nursing and Allied Health Literature) from the database inception to the search date by two authors independently. A repeated search was made in 7 January 2015 to find any additional literature.

The specific systematic review protocol was developed in accordance with PRISMA statement [18]. The literature searching method and included keywords were determined *a priori* by two authors.

### Study eligibility

Inclusion and exclusion criteria were determined before the database search was performed. Any studies that used arthroscopy as a sole method or as an adjuvant tool to diagnose or treat dysplastic hips and also satisfied any one or both of our purpose, that is (i) describing the arthroscopic finding and (ii) reporting the objective or subjective outcome, were included. Exclusion criteria were (i) Level V studies (case report and expert opinion), (ii) abstracts from conferences without full article availability, (iii) non-English language articles, (iv) non-human subjects and (v) including patient with age under 12 years. The review articles were also excluded but the bibliographies were hand searched to identify any additional references that fulfill our criteria.

All retrieved studies were first imported to Endnote X7 and duplicate studies were removed. The remaining studies were reviewed by two authors independently by titles and abstracts and the studies determined to be relevant from each authors were combined and full-text manuscripts was retrieved. The full reports were also reviewed by each authors independently for eligibility and any disagreements on inclusion were resolved by discussion. In the cases of articles with overlapping patient cohorts, a discussion was made between the reviewers to include the article which best fit to our purpose. The data of the resultant studies were extracted to prearranged summary tables.

## RESULT

**Fig. 1.**
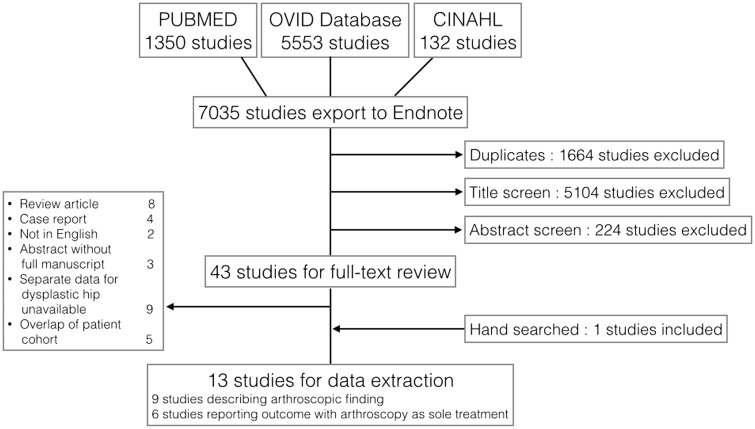
A flow diagram showing screening process.

Using the search terms described, 7035 studies were initially retrieved. Forty three studies progressed to full manuscript review. Thirteen studies from 11 institutions were selected for final data extraction (Fig. 1).

### Study characteristics

Of the 13 studies reviewed, nine studies described the arthroscopic findings. Two of these studies reported the outcomes of arthroscopic treatment [12, 19] and, combined with four additional studies [20–23], a total of six studies reported subjective and/or objective results. All studies were Level IV (Case series) studies and thus formal quality scoring for individual studies was not performed.

Two studies from one institution were included for review of arthroscopic findings as there was no overlap of the patient cohorts [12, 24]. Another institution first reported the arthroscopic findings and outcome [21] and later reported only the arthroscopic finding with a larger cohort [25] which overlap the patient with the previous study. Thus, the earlier study from this institution was included for the review of outcome and the later study was included for the review of arthroscopic findings.

The rationales for performing arthroscopy were described in six studies but were mostly ambiguous. Consistent indications included failure of response to conservative treatment, mechanical symptoms and positive physical examination findings. However, the nature of conservative treatment, duration, patient symptoms and physical examination used varied across the studies. Three studies which used arthroscopy as an adjuvant therapy with osteotomy used advanced radiographic exams to detect intra-articular pathologies before the arthroscopy and two of these studies used the findings of the radiographic exam as rationale for performing concurrent arthroscopy.

### Definition of dysplastic hip

The most common radiologic marker for diagnosis of dysplasia was lateral center edge angle (LCEA) as described by Wiberg. Dysplasia was defined as LCEA <20° and borderline dysplasia was defined as LCEA between 20° and 25° except in one institution [21, 25] which defined dysplasia as CE angle below 22° and borderline dysplasia as between 22° and 28° as reported by Massie and Howorth [26]. Other parameters used were increased acetabular index (>10°) and anterior center-edge angle <20°.

### Intra-articular findings

Six studies reported the arthroscopic findings observed while utilizing arthroscopy as an adjuvant tool while three studies were using arthroscopy as a sole treatment method. Overall, across the nine studies, there were a total of 538 hips from 525 patients. Two studies did not specify male to female ratio, and excluding these studies, female gender constituted the majority of the patients (85%). One study reported only borderline dysplasia and three studies included borderline dysplasia as a part of their patient cohorts (15%, 12.3% and 66.7%, respectively). While labrum condition was described in all studies, chondral lesions were reported in seven studies and LT lesions in five. Three studies reported the presence of synovitis and two studies reported loose bodies.

The prevalence for labrum tear across the study ranged from 56 to 100% with the overall prevalence of 77.3% (416 labrum tear in 538 hips). Five studies described the locations of the labrum tear which were mostly located in anterior or anterosuperior areas of the acetabular rim. Chondral damage was reported for acetabulum and femur separately in four studies and two studies reported the overall rate combined. Overall, cartilage lesions were more common on the acetabular side (range 59–75.2%) as compared with the femoral head side (range 11–32%). The cartilage damage was also more commonly located in anterior or anteriosuperior region both in the acetabulum and in the femur. The prevalence of LT tear varied significantly among the studies. Five studies reported prevalence of LT tear ranging from 0 to 71% with overall rate of 15.5% (51 LT tears in 330 hips).

Other findings included synovitis (reported in three studies with 2%, 52%, 69% prevalence, respectively) and loose body (reported in two studies with 8.2% and 17% prevalence) (Table I).

**Table I:** Arthroscopic findings of intra-articular pathologies in dysplastic hip

Study	Age (year)	Male %	No of hips (patients)	Borderline Dysplasia	Dysplasia definition	Findings	Special Notes
McCarthy and Lee [25], 2002	35 (12–58)	27%	170 (163)	15%	LCEA: mild 22–28°, moderate 16° to 22° Lateral roof arc 0° or upsloping	Labral tear 72% (93% at anterior) Ant acetabular chondral lesion 59% Ant femoral head chondral lesion 32% LT lesion: none Synovitis 69%	Exclude <2 mm joint space
Byrd and Jones [19], 2003	34 (14–64)	42%	48 (48)	66.7%	LCEA: borderline 20–25°, dysplastic <20°	Labral pathology 75% Chondral damage 60% LT tear 27% Loose body 17% Synovitis 2%	Arthritis in 9 hips
Girard [47], 2007	35 (20–59)	N/A	26 (26)	0%	LCEA <20°	Labral tear 62% (69% at superior aspect)	16 grade 3 and grade 4 OA by Mourgues and Patte’s criteria
Berton [48], 2010	34 (20–49)	N/A	18 (18)	0%	LCEA <20°	Labral tear 56% (70% at superior)	Include 3 grade 2, 1 grad 3 OA (Tonnis grade)
Ross [38], 2011	28 (12–47)	6.8%	73 (71)	12.3%	LCEA: borderline 20–25°, dysplastic <20° ACEA <20°	Labral tear or detachment 65.8% (81% at anterior) Acetabular cartilage lesion 67.1% (anterior 76% anterolateral 84%) Femoral chondromalacia 11% LT tear 30.1% Loose body 8.2% Synovitis 52%	5.5% had grade 2 OA (Tonnis grade)
Kim [49], 2011	40 (20–67)	16%	43 (40)	0%	LCEA 10° to 19°	Labral lesions 88.4%	13 grade 2 or 3 OA (Tonnis grade)
Fujii [50], 2011	40.2(13–64)	9%	121 (121)	0%	LCEA <20°	Labrum tear 81.8% Acetabular cartilage lesion 75.2% Femoral cartilage lesion 28.1%	56% had grade 2 and 23% grade 3 OA (K-L grade)
Domb *et al.* [12], 2013	20 (14–39)	18%	22 (22)	100%	LCEA: 18° to 25°	Labrum tear 100% Cartilage damage 86% LT tear 59%	Exclude Tonnis grade 2,3 OA,Legg-Calves-Perthes
Domb [51], 2014	21 (12–32)	25%	17 (16)	0%	LCEA 10° to 17°, ACEA 0° to 20°	Labral tear 76% (between 1–3’O clock) Acetablar chondral lesion 67% Acetabular and femoral chondral lesion 9.5% LT tear 71%	Exclude Tonnis grade 2,3 OA

LT, ligamentum teres; LCEA, laeral center-edge angle; ACEA, anterior center-edge angle; OA, osteoarthritis.

## OUTCOMES

All of the six studies which described the outcome of dysplastic hips treated without concurrent osteotomy were Level IV studies. Three studies described the result of only the borderline dysplasia and two studies reported result of dysplastic hips. One study included both categories and the outcome scores were reported separately. The minimum follow up period was 1 year in four studies and 2 year in two studies. Different outcome scores were utilized to assess patient outcome across the studies which limits former meta-analysis.

Of the four studies which reported the outcome of borderline dysplasia, three studies reported favorable outcomes whereas one study reported poor prognosis after labral debridement. One study emphasized the plication of capsule after intra-articular lesions were treated.

Ambivalent results were reported among the three studies which reported outcome of arthroscopic treatment for dysplasia. All three studies used arthroscopic debridement for labral tear but while two studies reported promising outcomes, one study reported the deterioration of functional scores after 2 years, in which 67% of the patients requiring reoperation. The study characteristics and outcomes are described in Tables II and III.

**Table II:** Epidemiology of arthroscopy used as sole treatment for dysplastic hip

Study	Study design	No of hips (pt)	Male:Female	Type of dysplasia included	Follow up length (month)	Arthroscopic treatment methods	Special note
McCarthy and Lee [21]	IV, Case Series	20 (19)	7:13	Borderline dysplasia	Minimum 24	Arthroscopic management (procedure not specified)	All patients were diagnosed borderline dysplasia with CE angle between 19° and 27°
Byrd and Jones [19]	IV, Case Series	48 (48)	20:24	67% Borderline 33% Dysplastic	27 (12–60)	Labrum tear -> Labral excision Unstable articular cartilage -> Chondroplasty Grade IV articular lesion -> Micro-fx LT disruption -> Debridement Loose body -> Removal Synovial disease -> Debridement	Each surgical procedure were dictated by the nature of the pathology encountered 9 hips had arthritis (grade unspecified)
Yamamoto *et al.* [22]	IV, Case Series	10 (10)	0:10	Dysplastic	96 (24–168)	Labrum tear -> partial limbectomy	4 hips with early arthritis
Parvizi *et al.* [20]	IV, Case Series	36 (34)	12:22	Dysplastic	40 (12–84)	Labrum debridement performed in all hips	Arthroscopy performed specifically for labrum repair and/or excision were followed 9 hips had early arthritis
Kalore and Jiranek [23]	IV, Prognostic study	50	6:44	Borderline dysplasia	33 (12–65)	Labrum tear -> repair (50%), debridement (50%)	Results were compared between borderline and adequate acetabular coverage, debridement versus repair
Domb *et al.* [12]	IV, Case Series	22 (22)	4:18	Borderline dysplasia	27.5 (17-39)	Labrum tear -> repair without detachment (21 hips) Lt tear -> debridement (1 hip) Unstable cartilage lesion -> chondroplasty Pincer lesion -> minimal rim resection	Capsular plication was done in all cases

**Table III.** Outcome of arthroscopy used as sole treatment for dysplastic hip

Study	Subjective outcomes	Improvement in outcome scores	Complications and subsequent operations	Conclusion
McCarthy and [21]	85% reported absence of preoperative hip pain		Subsequent arthroscopy for loose body: 1 Advance degeneration change: 3	Arthroscopy can relieve symptoms from lateral tear Overzealous labral removal should be avoided
Byrd and Jones [19]		Dysplastic group mHHS: 57 -> 83 Borderline dysplasia group mHHS: 50 -> 77	Arthroplasty: 2	Dysplasia is not a contraindication for arthroscopy Results of arthroscopy are dictated by type of pathology
Yamamoto *et al.* [22]	All patients satisfied 3 patients had mild or moderate dull pain	HHS: 64.5 -> 92.5	Lateralization of femoral head without symptom: 1	Arthroscopic partial limbectomy in a dysplastic hip does not lead to rapid progression of osteoarthritis
Parvizi *et al.* [20], 2009	Failed to control symptoms in 67%	Functional outcome (Short form 36, SUSHI) at 2 years: 76 Accelerated arthritis (39%) Migration of femoral head (36%)	Subsequent arthroscopy: 3 Periacetabular osteotomy: 6 Femoroacetabular osteoplasty: 7 THA: 3	Labral excision in symptomatic dysplasia can have detriemental effect
Kalore and Jiranek [23]	HOOS shows no significant difference between borderline and adequate coverage, labral debridement versus repair		Reoperation: 15	Reoperation rate is higher in patients with borderline acetabular coverage, Borderline dysplasia with labral debridement result in poor prognosis
Domb *et al.* [12]	VAS: 5.8 -> 2.9 Patient satisfaction: 8.4	mHHS: 69.0 -> 86.2 HOS-ADLS: 72.9 -> 89.6 HOS-SSS: 49.0 -> 77 NAHS: 68.6 -> 85.9	3 hips progress from Tonnis grade 0–1 Revision: 2	Mild dysplasia can be successfully treated with arthroscopy when the treatment aimed at instability

SUSHI, Super Simplified Hip Score; HOOS, Hip Dysfunction and Osteoarthritis Outcome Score.

## DISCUSSION

The shallow acetabulum in a dysplastic hip increases contact forces within the joint and is a common cause of osteoarthritis in young adults responsible for 20–40% of all symptomatic arthritis [27–33]. A recent prospective study of 720 participants showed significantly high rate of developing osteoarthritis in dysplastic hip with adjusted odd ratio of roughly 3–4 [34]. Several anatomical variants can occur as a result of altered hip biomechanics including elongation of the LT and hypertrophy of the actabular labrum and together with the shallow acetabulum and anterior migration of the femoral head, the injury of LT, labrum, or articular cartilage can be commonly witnessed [35–37]. In the current review, labral tears were observed in over three-quarters of the symptomatic dysplastic hips and also a high incidence of cartilage lesions and LT injuries was observed. However, it is uncertain whether the severity of dysplasia has the correlation with the incidence of the lesions as the indication for surgery and the prevalence rate varied across the studies. Only one study discussed this and reported that patients with higher grade dysplasia (LCEA < 15°) were associated with an increased prevalence and severity of the acetabular chondromalaica [38].

A number of studies have reported the intra-articular findings by direct visualization with arthrotomy. However, a previous systematic review revealed a substantially high rate of labral tear detection with the use of hip arthroscopy as compared with arthrotomy [39]; thus we chose to report only the studies using arthroscopy to explore hip joints. We do acknowledge, that the experience of the surgeons, the tools used (30° versus 70° telescope), and indication for surgery can influence the detection of the pathologic lesions. Nevertheless, most studies in this review reported both the injury nature and location of the labrum tear in detail and the prevalence rate was consistently high regardless of whether advanced radiographic examinations were used as part of the indication for arthroscopy.

The ultimate treatment for symptomatic dysplastic hip involves correction of the orientation and depth of the acetabulum. Number of pelvic osteotomy procedure has been proposed to achieve this which includes shelf osteotomy, periacetabular osteotomy (PAO) and Birmingham interlocking periacetabular osteotomy (BIPO). The review of the PAO shows improved pain and function at short to midterm follow up in majority of the hips with survival rate ranging 83–100% [17]. The recent publication reported the survival rate after BIPO to be as high as 89% in the patients under 20 years of age and 67% in the patient over 30 years at 20 year follow up [40]. However, these procedures require substantial time for rehabilitation and the result may vary depend on the experience of the surgeon. Also, the reported complications are common with major complications ranging from 6 to 37% [17]. The use of arthroscopy on dysplastic hip was traditionally viewed negatively. The symptoms associated with hip dysplasia are thought to originate from intra-articular damage secondary to structural deformities. The debridement of labrum has been suggested to cause progression of cartilage damage as the hypertrophied labrum commonly seen in dysplasia is likely to be a protective structure against micro-instability [9, 41]. Also, it has been suggested the pain relief from arthroscopic procedure may lead to ignorance of underlying abnormal mechanics and would lead to arthritis [11, 42]. The study from Ross *et al*. [24] indicates, failure to correct the pathoanatomy may result in suboptimal patient outcome.

However, the result of this study indicates that the published evidence for avoiding arthroscopy in dysplastic or borderline dysplastic hips is weak and although it should be approached carefully, the arthroscopic procedures may provide at least short term benefit. The conclusion derived in the study from Byrd and Jones [19] was that the results of arthroscopic surgery are dependent on the underlying intra-articular lesions and not the presence of acetabular dysplasia. Also, the study from Yamamoto *et al.* [22] reported that none of the patients had progressed to osteoarthritis over mean of 8 years after arthroscopic partial limbectomy in the patient with CE angle between 0° and 20°. The study of Kalore and Jiranek [23] reported arthroscopic debridement in borderline dysplasia resulted in a higher reoperation rate but there was no significant difference when the labrum was repaired as compared to hips with adequate acetabular coverage. Only one study reported unacceptably high failure rate which led to abandonment of the use of arthroscopy in dysplstic patients in their institution [20].

This is inconsistent with some of the case reports which reported catastrophic results after arthroscopic treatment. The study from Matsuda and Khatod [43] reported two cases where rapidly progressive osteoarthritis developed after arthroscopic labral repair. A similar result was reported by Mei-Dan *et al*. [44] in a patient with borderline dysplasia. Both studies suggested that increased instability after arthroscopy may have contributed to the rapid aggravation of arthritis. In such cases, the capsular plication as suggested by Domb *et al*. [12] could be utilized to attempt to prevent instability. Also, it should be noted that across the studies reviewed, the authors emphasized avoidance of over-zealous resection.

### Arthroscopy as an adjuvant treatment

The role of arthroscopy can expand to be an adjuvant tools with concomitant pelvic osteotomy procedures. However, the incidence of labral tear and its outcome after concurrent osteotomy is controversial. Domb *et al.* [45] described the benefit of hip arthroscopy prior to PAO to be (i) diagnosis of intra-articular pathology which could alter of the indication from PAO to arthroplasty and (ii) treatment of intra-articular pathology which could provide better surgical outcome. Despite the number of studies reporting favorable outcome of PAO with adjuvant arthroscopy, it is unclear whether the symptom improvement is a result of structural change or from treating intra-articular lesions with arthroscopy. Although we did not review this subject in this current review, we agree that cartilage damage can be more severe than presumed preoperatively with conventional imaging and pre-osteotomy arthroscopy in severe dysplastic hip may guide surgeons to determine the most appropriate operation.

### Limitation and future study

Despite the detailed systematic review of the outcomes of arthroscopic treatment for hip dysplasia, there are only a limited number of studies dealing with this subject and reporting with limited treatment option. Recent studies have reported better outcomes when the labrum is reconstructed or repaired when compared with labral debridement or resection. Also, reconstruction of LT has been reported to provide stability of the hip joint [46]. As none of the studies reviewed included reconstruction of the labrum or LT, it is uncertain whether these procedures could provide benefit when treating a dysplastic hip. Another limitation is that most of the studies reviewed reported relatively short term outcome and the result after long term follow up is limited. Finally, none of the studies has reported the result of a control group, with the same pathology treated non-operatively, thus whether arthroscopy is responsible for short term benefits or aggravations remain unanswered. Nevertheless, further studies should be performed under the boundaries of strict ethics.

## CONCLUSION

This systematic review reports that the intra-articular pathology in symptomatic dysplastic hip is extremely common with high incidence of labral tear, cartilage damage and LT injury. The short term result of arthroscopic treatment for this pathology seems to be beneficial in borderline dysplasia but the effect of it in moderate dysplasia is unclear and need careful approach. Special question raised from this study is the long term result of the previously published reports and the effect of LT or labrum reconstruction on this subjects.

## FUNDING

This work received no funding.

## CONFLICT OF INTEREST STATEMENT

None declared.
